# Psychometric properties of Postpartum Partner Support Scale—Persian version

**DOI:** 10.1002/nop2.806

**Published:** 2021-02-19

**Authors:** Zahra Eslahi, Zainab Alimoradi, Nasim Bahrami, Chung‐Ying Lin, Mark D. Griffiths, Amir H Pakpour

**Affiliations:** ^1^ Students Research Committee School of Nursing & Midwifery Qazvin University of Medical Sciences Qazvin Iran; ^2^ Social Determinants of Health Research Center Research Institute for Prevention of Non‐Communicable Diseases Qazvin University of Medical Sciences Qazvin Iran; ^3^ Department of Rehabilitation Sciences Hong Kong Polytechnic University Hong Kong Hong Kong; ^4^ Institute of Allied Health Sciences and Departments of Occupational Therapy and Public Health, National Cheng Kung University Hospital, College of Medicine Taiwan National Cheng Kung University Tainan Taiwan; ^5^ International Gaming Research Unit Psychology Department Nottingham Trent University Nottingham UK; ^6^ Department of Nursing School of Health and Welfare Jönköping University Jönköping Sweden

**Keywords:** partner support, postpartum, postpartum partner support scale, psychometric evaluation, social support

## Abstract

**Aim:**

The aim of the present study was to translate the Postpartum Partner Support Scale (PPSS) into Persian and evaluate its psychometric properties among postpartum women.

**Design:**

A total of 248 women aged 18–39 years participated in this psychometric study. The PPSS was translated into Persian using a forward‐backward method. Confirmatory factor analysis (CFA) and Rasch model analysis were used to assess the psychometric properties of the PPSS. In addition, the Edinburgh Postpartum Depression Scale (EPDS) was completed simultaneously to assess the construct validity. Internal consistency of the questionnaire was assessed by calculating the Cronbach's alpha coefficient and corrected item‐total correlation.

**Results:**

The unidimensionality of the PPSS was supported in both CFA and Rasch analysis. The PPSS had a significant negative association with EPDS (r = −0.39 *p* < .001). The scale had excellent internal consistency (Cronbach's alpha = 0.94) and the correlation between items and total score was satisfactory.

**Conclusion:**

The Persian version of PPSS with 20 items is a valid and reliable scale to assess postpartum support.

## 1 INTRODUCTION

Postpartum women undergo many unintended changes in various aspects concerning their quality of life (Da Costa et al., 2006; Shaw, Levitt, Wong, Kaczorowski, & Group, 2006; Torkan et al., 2009; Torshizi & Sharifzadeh, 2013). Psychological changes including postpartum blues, depression and psychosis (Akbarzadeh et al., 2012; Schiller et al., 2015), physical changes including fatigue, headache, low back pain, abdominal pain, vaginal/intercourse pain, constipation and/or haemorrhoids, urinary and/or excretory problems, and breast pain (Schaffir et al., 2018; Webb et al., 2008) are experienced during postpartum. Webb et al. (2008) reported approximately 70% of women experience at least one of the aforementioned physical problems during the postpartum period (Webb et al., 2008). Although postpartum physical problems are often considered transient and minor, they have a strong association with women's functional problems and poor health outcomes (Webb et al., 2008). The responsibility for taking care of the baby and breastfeeding makes postpartum women tired. Psychological problems, concerns about appearance and neonatal illness are other issues that need support. Low levels of social support decrease women's self‐efficacy in protecting and caring for infants (Gao et al., 2014; Shorey et al., 2014).

Postpartum mothers’ health has received much attention by researchers across various cultures (Cheng & Pickler, 2009). For example, in Chinese culture, women should be supported and assisted in their daily activities for a month after childbirth (Chen & Wang, 2000). Because of the support and assistance provided by partner during this month, mothers of newborn babies can recover from postpartum fatigue and regain lost energy (Chen & Wang, 2000). Chein et al. (2009) found that parturient women who received this care experienced less severe postpartum physical symptoms, decreased chances of postpartum depression and overall improved physical health (Chien et al., 2006). Emotional and practical support during the postpartum period also increases maternal self‐efficacy in infant care practices such as breastfeeding and increases the duration of breastfeeding (Leahy‐Warren et al., 2012; Rempel et al., 2017).

Fatigue, postpartum blues, postpartum stress, anxiety and depression are all conditions that require social support (Hopkins & Campbell, 2008). Social support is defined as the mental sense of belonging, acceptance, popularity and worthiness and is coupled with the possibility of receiving help in an emergency (Uchino, 2004). Social support includes the help and comfort that individuals expect from a stable and ongoing relationship with a person or group (Cutrona, 1996). Interpersonal social support exchanges between members of specific social networks are in the form of two‐way and informal relationships that are usually formed spontaneously. It has a functional domain (perceived social support) and a structural domain (social network size) (Lakey & Cohen, 2000). Based on House’s (1987) theory, social support comprises the functional content of relationships and can be classified into four groups of supportive behaviours (i.e. emotional support, material support, information support and evaluation support). These four functions of protection are conceptually different, but in practice they are not independent of each other (House, 1987; House et al., 1988).

The arrival of a new member in the family causes dynamic changes in the family that predispose the physical and emotional vulnerability of family members (Orshan, 2008). The receiving of social support is one of the factors that plays a very important role in reducing maternal vulnerability and stress (Mann et al., 2010). In general, evidence suggests that social support during pregnancy and after childbirth has a protective role. Pregnant and parturient women need the support of family members, husbands, friends and health professionals (Hodnett & Fredericks, 2003). These individuals can provide psychological support to mothers as well as providing information (Gooding et al., 2011). Social support is the strongest factor in successfully coping with stressful situations such as postpartum changes among mothers (Asgari & Sharaf Aldin, 2008). In essence, social support comprises an interpersonal relationship that provides psychological assistance when needed (Gooding et al., 2011).

Consequently, postpartum support can improve maternal and infant well‐being and health by assisting the parturient mother in transition into motherhood (Stapleton et al., 2012). Among the sources of support, the woman's partner has been identified as the most important source of support for a parturient woman (Heydari et al., 2009). This is because during this period, women are deprived of their former social networks and relationships and rely more on their partner's support (Rowe & Fisher, 2010). Previous systematic reviews confirmed the strong predictive role of partner support for postpartum depression and underline the importance of the social environment in which women live (Dennis & Dowswell, 2013; Fisher et al., 2012; Martini et al., 2015; Yim et al., 2015).

## 2 BACKGROUND TO THE PRESENT STUDY

Investigating different aspects of partner support in the postpartum period requires a specific psychometric instrument, and Postpartum Partner Support Scale (PPSS) is one of the best. The PPSS developed by Dennis et al. (2017) in Canada has strong psychometric properties (Dennis et al., 2017). However, to the best of the present authors’ knowledge, the PPSS has never been translated into Persian. Given the importance of the postpartum period in the physical, mental and quality of life of newborn women and their infants, using the validated Persian PPSS will help healthcare providers understand the role of partner support, an important source of support during postpartum. Therefore, the present study aimed to translate and evaluate the psychometric properties of the PPSS.

## 3 METHODS

### 3.1 Study design

This psychometric study was conducted between January and July 2019. The study protocol was approved by the regional Ethics Committee of Biomedical researches approved the project with decree code of IR.QUMS.REC.1397.120. Informed written consent was obtained from the participants after explaining the purpose of the study, and the privacy and confidentiality of the information.

### 3.2 Participants and sampling

Two hundred and seventy parturient women referred to urban comprehensive health centres for their second or third postpartum care participated in the study. Secondary postpartum care is provided routinely between 12–15 days postpartum and tertiary postpartum care 40–45 days postpartum at comprehensive health centres in Iran.

Sampling was done in two stages. In the first stage, health centres were considered as clusters and two health centres were selected from each of the five urban geographical areas in the north of Iran using a random cluster sampling. In the second stage, simple random sampling was used. To do so, in each of the health centres’ client lists, 25 individuals were invited to participate in the study. Inclusion criteria included being primiparous, having a healthy baby and living with a partner. Participants were excluded if they had a history of physical and mental illness and the experience of pregnancy and childbirth complications, such as preeclampsia and premature labour.

### 3.3 Sample size estimation

Considering that the main statistical analysis in this study was to evaluate the construct validity of the PPSS using CFA, the minimum required sample size based on the literature is 200–250 participants (Kline, 2015). According to the rules of thumb, CFA requires the estimated the sample size to comprise a minimum of 10 participants per item (Myers et al., 2011). In addition, the recommended sample size for Rasch analysis utilizing a five‐point Likert scale is 250 people (Embretson & Reise, 2000; Reeve & Fayers, 2005). Altogether, considering that the PPSS comprises 20 items responded to on a four‐point Likert scale, a total of 250 individuals were adequate for carrying out the analyses in the present study.

### 3.4 Measures

#### 3.4.1 Demographic and obstetric characteristics

The survey included questions regarding age, education level, occupation of the woman and her partner, economic status of family, gestational age at birth, baby's birth weight, gender and presence of underlying birth problems.

#### 3.4.2 Postpartum Partner Support Scale (PPSS)

The PPPS was developed by Dennis et al. and comprises 20 items on a four‐point Likert scale ranging from 1 (*strongly disagree*) to 4 (*strongly agree*). Scores range from 20–80. Higher scores indicate more postpartum partner support. PPSS was developed based on the theoretical model of social relationships and functional components of social support. In the present study, the use of the EPDS was utilized to test construct validity (i.e. the greater the postpartum partner support, the lower the depression). Psychometric evaluation including content validity, internal consistency, exploratory factor analysis, predictive validity and construct validity of original scale was assessed. Internal reliability using Cronbach's alpha for the scale is excellent (0.96) (Dennis et al., 2017).

#### 3.4.3 Edinburgh Postpartum Depression Scale (EPDS)

This 10‐item scale was designed to screen postpartum depression six weeks after delivery. EPDS scores vary between 0–30. Acquiring a score of 12 and above is considered as postpartum depression (Cox et al., 1987). Psychometric evaluation of the Persian version of this was confirmed in 2015 by Golzar et al, (Ahmadi kani Golzar & GoliZadeh, 2015). Measures used for this study are provided in File S1.

### 3.5 Translation process

After acquiring required permissions, the PPSS was translated into Persian using the forward‐backward method (Annette et al., 1994). Initially, the English version of the scale was translated into Persian by two translators who were native Persian speakers and who had sufficient experience in translating English texts. The initial translations were then combined into a single translation. At this stage, the newly translated version was reviewed and compared by experts. Any translation problems or language ambiguity were amended and merged. This agreed Persian version was then backward translated into English. This back‐translated English version was sent to and verified by the original developers of the PPSS.

### 3.6 Statistical analysis

Data were analysed using three different statistical packages: SPSS version 24 (IBM Corp, New York, USA), MPLUS version 7.0, and WINSTEPS 4.0.1 (Chicago, IL). Raw data used in statistical analysis are provided in File 2. Data are presented means (with SDs) for continuous data, and frequencies and percentages for categorical data. To assess psychometric properties of the PPSS, both classic test theory (CTT) and Rasch methods were used on the data. Within the framework of the CTT, possible floor and ceiling effects (<20% of participants scoring the lowest or highest possible PPSS total score (Jette et al., 2005)) were assessed, internal consistency was assessed utilizing Cronbach's alpha coefficient. Cronbach's alpha values higher than 0.7, indicated acceptable internal consistency. The corrected item‐total correlation was calculated to ensure that each item was well connected with the entire concept and values above 0.4 are acceptable.

Confirmatory factor analysis (CFA) using diagonally weighted least squares (WLSMV) estimator was conducted to examine the unidimensional structure of the PPSS. Model fit was evaluated using a series of fit indices including a non‐significant χ^2^ test, comparative fit index (CFI) >0.90, Tucker–Lewis index (TLI) >0.90, root mean square error of approximation (RMSEA), and standardized root mean square residual (SRMR) ≤0.08 (Brown, 2015). Factor loadings derived from CFA were used to calculate average variance extracted and composite reliability. Average variance extracted >0.5 and composite reliability >0.7 were considered acceptable.

Rasch analysis was performed using the Rasch partial credit model in the Winsteps software (version 3.75.0). The unit of *logit* was calculated for each item to report item difficulty, with higher logit values representing more difficult item. Item fit statistics was assessed using the information‐weighted fit statistic (infit), mean square (MnSq) and outlier‐sensitive fit statistic (outfit) MnSq. Values between 0.5–1.5 are interpreted as good fit. Items with low fit (i.e., MnSq <0.5) and high fit (i.e., MnSq >1.5) are considered as redundant and out‐of‐concept concept (Khan et al., 2013). Item reliability and person reliability were also calculated for the PPSS. Values with higher than 0.70 are considered acceptable. Item separation and person separation indices were conducted with a value >2.0 examining how well the participants can be discriminated (person separation index) and how well the items can be discriminated (item separation index).

Construct validity was also assessed by examining the Pearson correlation coefficients between the PPSS and EPDS. Based on Walker and Almond's (2010) guidelines, correlation coefficients of 0.15–0.3 are interpreted as weak, 0.3–0.59 moderate, and 0.6–1.0 strong in social sciences research.

## 4 RESULTS

### 4.1 Description of demographic characteristics

The mean ages of the women and their partners were 27.84 and 32.64 years, respectively. Most of the participants (81%) were housewives. The mean gestational age of women was 38.86 weeks, and the mean birthweight of their neonates was 3,407.49 grams. The mean participant scores on the EPDS and PPSS were 6.95 and 64.32, respectively. Table 1 shows the demographic characteristics of the participants.

**TABLE 1:** Demographic characteristics of participants

Quantitative variables	Mean (*SD*)
Women's age (year)	27.84 (4.72)
Partner's age (year)	32.64 (5.32)
Gestational age at delivery (weeks)	32.86 (1.3)
Neonate's birth weight	3,407.49 (1739.44)
EPDS (0–26)	6.95 (5.32)
PPSS (21–80)	64.32 (10.45)

Qualitative variables	No (%)
Educational status	
Elementary school	9 (3.6)
Guidance school	28 (11.3)
High school and diploma	112 (45.2)
Academic	99 (39.9)
Job	
Housewife	201 (81)
Employee	46 (19)
Neonate's gender	
Boy	116 (46.8)
Girl	132 (53.2)

### 4.2 Psychometric properties

There were no floor (0.4%) or ceiling (2.0%) effects for the PPSS. The Cronbach's alpha was excellent (0.94). None of the items yielded corrected item‐total correlations below 0.40 with values ranging from 0.49–0.82. Table 2 shows the results of item‐level psychometric properties. The results of the CFA showed that the single‐factor model provided an excellent fit (χ^2^ = 420.75, *df* = 170, *p* <.01; RMSEA= 0.077; CFI = 0.929; TLI = 0.919), and all the estimated parameters were statistically significant (*p* < .01). Moreover, each item saturated in its corresponding scale (Table 3).

**TABLE 2:** Psychometric properties of the Postpartum Partner Support Scale (PPSS) at item level

	Analyses from classical test theory			Analyses from Rasch		
Item	Factor loading	Item‐total correlation	Cronbach's alpha if item excluded	Infit MnSq	Outfit MnSq	Difficulty
1	0.66	0.64	0.94	0.85	0.80	−0.76
2	0.68	0.66	0.94	1.07	1.08	−0.39
3	0.71	0.70	0.94	0.85	0.77	−0.59
4	0.72	0.70	0.94	0.93	1.08	−0.57
5	0.62	0.60	0.94	1.03	0.97	−0.85
6	0.82	0.79	0.94	0.73	0.63	−0.10
7	0.77	0.75	0.94	0.77	0.74	0.12
8	0.78	0.76	0.94	0.71	0.94	−0.24
9	0.75	0.72	0.94	0.81	0.82	−0.23
10	0.64	0.62	0.94	1.38	1.30	0.40
11	0.79	0.78	0.94	0.68	0.60	−0.35
12	0.84	0.82	0.94	0.53	0.56	−0.46
13	0.50	0.49	0.94	1.49	1.35	0.92
14	0.49	0.49	0.96	1.09	1.19	1.90
15	0.78	0.76	0.94	0.67	0.67	−0.15
16	0.79	0.77	0.94	0.71	0.65	0.10
17	0.80	0.77	0.94	0.81	0.63	−0.71
18	0.82	0.80	0.94	0.56	0.54	0.02
19	0.76	0.73	0.94	0.70	0.66	−0.24
20	0.83	0.80	0.94	0.69	0.71	−0.80

**TABLE 3:** Psychometric properties of the Postpartum Partner Support Scale at scale level

Psychometric testing	PPSS	Suggested cut‐off
Cronbach's alpha	0.94	>0.7
Confirmatory factor analysis		
χ^2^ (*df*)	420.75 (170)*	Non‐significant
Comparative fit index	0.929	>0.9
Tucker‐Lewis index	0.919	>0.9
Root mean square error of approximation	0.077	<0.08
Standardized root mean square residual	0.043	<0.08
Average Variance Extracted	0.53	>0.5
Composite Reliability	0.95	>0.6
Item separation reliability from Rasch	0.99	>0.7
Item separation index from Rasch	9.0	>2
Person separation reliability from Rasch	0.91	>0.7
Person separation index from Rasch	3.17	>2

Note

* *p* <.001.

The results of the Rasch analysis are summarized in Table 3. The most difficult item was Item 14 (*“Provides me with feedback on how I am doing”*; 1.90 logit) and the easiest item was Item 5 (*“Makes me feel that I'm a good mother”*: −0.85 logit). In addition, all items fitted well on the underlying construct of Postpartum Partner Support (infit MnSq ranged 0.53–1.49; outfit MnSq ranged 0.54–1.35). There was a moderate significant (negative) correlation between the scores obtained from the two PPSS and EPDS scales (r = −0.39 *p*<.001).

## 5 DISCUSSION

The postpartum period for women especially among those undergoing their first maternal experience is a time of significant psychological, physical and social change that affects their quality of life (Hill et al., 2006). Assessing postpartum social support is an important and valuable issue that can affect mothers' physical and mental health. In order to assist postpartum women in overcoming difficulties due to delivery, it is important to improve their postpartum support. Therefore, the present study translated and evaluated the psychometric characteristics of the PPSS among Persian‐speaking women using CTT and Rasch model analysis. The two types of psychometric theories were used because CTT is the most commonly used approach for assessing psychometric properties and Rasch analysis provides additional insightful psychometric information. More specifically, CTT uses observed scores that are computed by summing up participant's item responses and estimates a true value of a given outcome. Consequently, CTT has the advantage of easy understanding. However, Rasch model analysis focuses on the differentiation between scales and participants. That is, Rasch model analysis has the advantage of sample‐free characteristic. More specifically, results from CTT substantially depend on the tested sample's characteristics; Rasch results do not have such problems because they generate item difficulty (i.e., whether the item makes the participant report low or high scores irrespective of their ability) and person ability (i.e., whether the participant has the ability to get low or high scores irrespective of the item's’ difficulty). With the separation of item and person, the psychometric properties derived from Rasch analysis are independent from tested sample's characteristics (Bond et al., 2020).

Using both CTT and Rasch approaches, the findings of the present study demonstrated that the Persian version of PPSS has unidimensional structure and good internal consistency. The undimensionality was similar to the English version developed by Dennis and colleagues (Dennis et al., 2017). In addition, the Persian version of PPSS had desirable reliability (internal consistency and separation reliability), construct validity and construct validity. Although Dennis et al. found that PPSS is a valid and reliable scale, they strongly suggested validating the PPSS in other cultures to expand the PPSS psychometric properties (Dennis et al., 2017). Therefore, this study is the first to investigate and confirm the psychometric properties of PPSS in Persian culture.

In the present study, construct validity was assessed using the EPDS. As expected, there was a significant moderate and negative correlation between the EPDS and PPSS. This means that with increased partner support in the postpartum period, postpartum depression decreases. This finding is consistent with the findings by Dennis et al. (2017) that the PPSS was inversely related to postpartum depression and anxiety (Dennis et al., 2017). In addition, many previous studies have shown that social support during postpartum period, irrespective of the measurement scale and the source of support, reduces postpartum blues and depression (Akbarzadeh et al., 2012; Cooper et al., 1999; Gao et al., 2014; Heidari & Hamooleh, 2011; McNatt & Freston, 1992; Najafi et al., 2006; Reid & Taylor, 2015).

Given the favourable psychometric properties of PPSS, this is valid and reliable scale for assessing the status of partner support in the postpartum period. Therefore, Persian‐speaking healthcare providers can use it when providing postpartum care. By identifying women at risk of unfavourable postpartum partner support, counselling with their partners can be planned, because social support in the postpartum period has a significant relationship with the mental health of newborn women (Dennis et al., 2017). In addition, this scale can be used to assess the effectiveness of social support‐promoting interventions in the postpartum period.

### 5.1 Strength and limitations

Using two types of psychometric methods, including CTT and Rasch analysis, to evaluate psychometric properties of the PPPS strengthened the results. The use of CTT analysis makes the results easier for health service providers to interpret (Chang et al., 2015). But calculating reliability based on item and participant, eliminating reliability dependency in the sample, and assessing measurement invariance for items are among the advantages of Rasch analysis (Chang et al., 2014). Therefore, the results of these two methods strongly support the validity of the psychometric properties of the Persian version of PPSS.

There are some limitations to the present study. First, both the PPSS and EPDS are self‐report scales. Therefore, issues concerning social desirability, recall biases, and common method variance might exist. Future studies may consider using different measures of depression (e.g., Hamilton Depression Rating Scale) to avoid the problems resulting from self‐report. Second, the sample was recruited from the same area of Iran. Therefore, the generalizability of the study's findings is restricted.

## 6 CONCLUSION

Based on the findings of the present study, the Persian version of PPSS has acceptable validity and reliability for use in the Persian population. Coupling the relative brevity of the PPSS (i.e., 20 items) with its strong psychometric properties, Persian‐speaking healthcare providers can use the PPSS quickly and with high efficiency. In relation to the contribution to nursing science, the scale will be of practical help. The postpartum period is a critical period in terms of physical, psychological and social health for the new mother. The spouse is among the most important and accessible sources of support during this time. Assessing the status of spouse support at postpartum visits will help healthcare providers in Persian‐speaking countries to design and provide appropriate and timely educational and counselling programs for the spouse to improve overall health for mothers and their newborn babies.

## CONFLICT OF INTEREST

None to declare.

## AUTHOR CONTRIBUTIONS

ME, ZA and AHP: Study design, data analysis and wrote manuscript. CYL, NB and MDG: Revised the manuscript for important intellectual content. All authors read and approved the final manuscript.

## Supporting information

Supplementary Material
