# Supplementary material for: Psychometric properties of Postpartum Partner Support Scale—Persian version
Source: Nurs Open. 2021 Feb 19;8(4):1688–95. doi: 10.1002/nop2.806 (PMC8186706; doi:10.1002/nop2.806)
Supplement: Supplementary file 1 — Supplementary Material [file NOP2-8-1688-s001.docx]

| **Postpartum Partner Support Scale (PPSS)** | | | | |
| --- | --- | --- | --- | --- |
| Below is a series of statements about your spouse/partner and the support provided after the birth of your baby. Please indicate which number comes closest to how you have been feeling during the past 4 weeks. | | | | |
|  | Strongly disagree | Disagree | Agree | Strongly Agree |
| My husband respects the decisions I take as a mother. | 1 | 2 | 3 | 4 |
| My husband is always with me when I need him. | 1 | 2 | 3 | 4 |
| My husband helps me take care of our child | 1 | 2 | 3 | 4 |
| My husband encourages me to ask for help when needed | 1 | 2 | 3 | 4 |
| My husband agrees with how I care for our baby. | 1 | 2 | 3 | 4 |
| My husband listens to my concerns. | 1 | 2 | 3 | 4 |
| My husband offers helpful suggestions to help me overcome my concerns | 1 | 2 | 3 | 4 |
| My husband cares about me and how I do. | 1 | 2 | 3 | 4 |
| My husband increases my confidence to be a good mother. | 1 | 2 | 3 | 4 |
| My husband participates in household chores. | 1 | 2 | 3 | 4 |
| Encourages me when worried about my mother's duties and responsibilities. | 1 | 2 | 3 | 4 |
| My husband helps me cope with difficult situations. | 1 | 2 | 3 | 4 |
| My husband gives me feedback on how I am doing. | 1 | 2 | 3 | 4 |
| My husband opposes me more since the birth of our baby. | 1 | 2 | 3 | 4 |
| My husband helps me solve any problems that I have. | 1 | 2 | 3 | 4 |
| My husband helps me find answers to my questions. | 1 | 2 | 3 | 4 |
| I feel that I'm getting better after talking to my husband. | 1 | 2 | 3 | 4 |
| My husband makes me feel that I can count on his help when needed. | 1 | 2 | 3 | 4 |
| My husband helps me see the positive aspects of the issues. | 1 | 2 | 3 | 4 |
| In general, I am satisfied with the support of my husband. | 1 | 2 | 3 | 4 |

**Edinburgh Postnatal Depression Scale (EPDS)**

Since you have recently had a baby, we want to know how you feel. Please place a CHECK MARK (✔) on the blank by the answer that comes closest to how you have felt IN THE PAST 7 DAYS—not just how you feel today

**1. I have been able to laugh and see the funny side of things:**

As much as I always could ____ (0)

Not quite so much now ____ (1)

Definitely not so much now ____ (2)

Not at all ____ (3)

**2. I have looked forward with enjoyment to things:**

As much as I ever did ____ (0)

Rather less than I used to ____ (1)

Definitely less than I used to ____ (2)

Hardly at all ____ (3)

**3. I have blamed myself unnecessarily when things went wrong:**

Yes, most of the time ____ (3)

Yes, some of the time ____ (2)

Not very often ____ (1)

No, never ____ (0)

**4. I have been anxious or worried for no good reason**:

No, not at all ____ (0)

Hardly ever ____ (1)

Yes, sometimes ____ (2)

Yes, very often ____ (3)

**5. I have felt scared or panicky for no good reason:**

Yes, quite a lot ____ (3)

Yes, sometimes ____ (2)

No, not much ____ (1)

No, not at all ____ (0)

**6. Things have been getting to me:**

Yes, most of the time I haven’t been able to cope at all ____ (3)

Yes, sometimes I haven’t been coping as well as usual ____ (2)

No, most of the time I have coped quite well ____ (1)

No, I have been coping as well as ever ____ (0)

**7. I have been so unhappy that I have had difficulty sleeping:**

Yes, most of the time ____ (3)

Yes, sometimes ____ (2)

No, not very often ____ (1)

No, not at all ____ (0)

**8. I have felt sad or miserable:**

Yes, most of the time ____ (3)

Yes, quite often ____ (2)

Not very often ____ (1)

No, not at all ____ (0)

**9. I have been so unhappy that I have been crying:**

Yes, most of the time ____ (3)

Yes, quite often ____ (2)

Only occasionally ____ (1)

No, never ____ (0)

**10. The thought of harming myself has occurred to me:**

Yes, quite often ____ (3)

Sometimes ____ (2)

Hardly ever ____ (1)

Never ____ (0
